# Supplementary material for: Development of Durum Wheat Breads Low in Sodium Using a Natural Low-Sodium Sea Salt
Source: Foods. 2020 Jun 5;9(6):752. doi: 10.3390/foods9060752 (PMC7353580; doi:10.3390/foods9060752)
Supplement: Supplementary file 1 [file foods-09-00752-s001.pdf]

Table S1. Colour parameters of the bread samples produced using different types and levels of sea salt during storage (data are means ± standard deviations). Three bread loaves were collected at each sampling.

Days of storage	Type	Crumb *L**	Crumb *a**	Crumb *b**	Crust *L**	Crust *a**	Crust *b**
0	Control A	70.36±0.44 abc	-3.24±0.16 cdefg	17.19±0.44	47.35±1.97 abcd	13.10±0.53 abc	26.85±0.60
	Control B	70.40±2.04 abc	-3.51±0.06 fg	18.97±0.72	49.54±2.76 abc	12.51±0.55 abcd	28.14±1.30
	1A	72.43±0.90 abc	-3.55±0.04 fg	19.35±0.36	48.32±1.43 abcd	13.70±1.19 a	28.93±0.30
	1 B	71.30±1.21 abc	-3.47±0.15 efg	20.18±0.59	37.82±2.60 e	12.42±0.20 abcd	21.77±1.37
	2 A	70.14±0.84 abc	-3.37±0.15 efg	18.73±0.37	41.13±1.98 cde	12.10±1.01 abcde	22.81±0.90
	2 B	71.18±1.33 abc	-3.41±0.05 efg	18.94±0.67	43.22±1.38 bcde	11.86±0.99 abcde	23.34±1.46
15	Control A	70.76±3.08 abc	-3.13±0.07 abcdefg	18.2±0.60	45.39±2.13 bcde	12.75±0.79 abcd	25.96±1.91
	Control B	71.55±1.22 abc	-3.33±0.28 defg	20.93±1.40	46.42±1.82 bcde	13.16±0.39 ab	27.29±1.46
	1A	71.79±0.59 abc	-3.61±0.09 g	21.01±0.81	47.59±1.18 abcd	12.29±1.00 abcde	25.77±1.88
	1 B	71.51±1.00 abc	-3.41±0.12 efg	20.45±0.83	42.27±2.56 bcde	11.90±0.50 abcde	23.3±1.67
	2 A	72.61±0.33 abc	-3.39±0.19 efg	21.62±1.85	43.11±3.29 bcde	11.30±0.58 abcde	22.03±1.42
	2 B	70.13±0.61 abc	-3.42±0.27 efg	20.46±1.05	42.55±0.61 bcde	10.38±1.32 bcde	22.31±0.99
30	Control A	74.71±2.83 a	-2.56±0.09 ab	20.34±1.51	50.33±1.84 abc	12.78±0.76 abcd	28.54±0.72
	Control B	74.57±1.46 ab	-3.00±0.05 abcdefg	20.32±0.20	50.75±2.81 ab	11.15±0.66 abcde	27,00±0.03
	1A	70.83±1.22 abc	-3.04±0.19 abcdefg	20.41±1.17	46.27±0.60 bcde	11.60±1.71 abcde	25.93±1.21
	1 B	70.31±1.57 abc	-2.84±0.09 abcde	20.55±2.12	45.39±2.82 bcde	9.91±0.71 de	20.67±3.93
	2 A	71.94±0.68 abc	-3.19±0.16 bcdefg	18.93±0.31	43.07±1.41 bcde	10.57±0.66 bcde	22.43±0.69
	2 B	71.63±0.85 abc	-2.93±0.37 abcdef	21.86±1.70	44.35±1.51 bcde	9.97±0.88 cde	23.37±2.29
60	Control A	69.39±1.68 bc	-2.66±0.28 abc	19.76±0.42	45.44±1.70 bcde	11.94±0.58 abcde	24.81±1.71
	Control B	72.52±2.95 abc	-3.23±0.23 cdefg	21.15±1.59	47.84±1.59 abcd	12.28±0.26 abcde	26.49±0.82
	1A	71.51±1.62 abc	-3.07±0.04 abcdefg	20.86±0.58	44.14±2.13 bcde	11.86±0.76 abcde	24.88±1.89
	1 B	71.95±2.46 abc	-3.21±0.28 bcdefg	20.61±0.52	39.26±1.60 de	11.15±0.37 abcde	23.15±0.64
	2 A	68.19±0.78 c	-2.83±0.23 abcde	20.91±0.41	42.65±4.60 bcde	10.70±1.26 abcde	23.57±1.74
	2 B	69.72±1.34 abc	-2.98±0.22 abcdefg	21.65±0.67	44.42±2.48 bcde	10.14±0.60 bcde	22.26±0.86
90	Control A	72.55±0.58 abc	-2.51±0.15 a	20.61±0.72	56.52±3.29 a	9.17±0.11 e	26.82±0.17
	Control B	74.40±1.75 ab	-3.32±0.14 defg	22.11±0.90	47.00±5.69 bcde	10.91±1.44 abcde	24.97±0.47
	1A	71.64±1.66 abc	-3.05±0.25 abcdefg	20.69±0.50	46.36±2.75 bcde	12.56±0.78 abcd	26.86±1.94
	1 B	71.39±0.55 abc	-3.05±0.10 abcdefg	21.64±0.65	41.43±2.31 bcde	10.23±0.23 bcde	21.68±0.54
	2 A	69.87±3.25 abc	-2.70±0.20 abcd	21.07±0.88	43.33±2.05 bcde	9.74±0.62 de	21.61±1.32

2 B	69.94±1.21 abc	-2.68±0.04 abcd	21.48±0.67	42.41±5.10 bcde	10.96±1.41 abcde	21.92±3.23

Different letter in the same column indicates significant difference ($p \leq 0.01$).
